# Phragmén’s voting methods and justified representation

**DOI:** 10.1007/s10107-023-01926-8

**Published:** 2023-03-06

**Authors:** Markus Brill, Rupert Freeman, Svante Janson, Martin Lackner

**Affiliations:** 1 TU Berlin, Berlin, Germany; 2 University of Warwick, Coventry, UK; 3 University of Virginia, Charlottesville, VA USA; 4 Uppsala University, Uppsala, Sweden; 5 TU Wien, Vienna, Austria

**Keywords:** 91B14

## Abstract

In the late 19th century, Swedish mathematician Edvard Phragmén proposed a load-balancing approach for selecting committees based on approval ballots. We consider three committee voting rules resulting from this approach: two optimization variants—one minimizing the maximum load and one minimizing the variance of loads—and a sequential variant. We study Phragmén ’s methods from an axiomatic point of view, focusing on properties capturing proportional representation. We show that the sequential variant satisfies *proportional justified representation*, which is a rare property for committee monotonic methods. Moreover, we show that the optimization variants satisfy *perfect representation*. We also analyze the computational complexity of Phragmén ’s methods and provide mixed-integer programming based algorithms for computing them.

## Introduction

While most of the social choice literature is focused on single-winner scenarios, recent years have witnessed an increasing interest in *committee voting rules* (e.g., [19, 21, 31, 60]). In this setting, a fixed-size subset of alternatives has to be selected based on the preferences of a group of voters. In this paper, we assume that the preferences of individual voters are given by *approval ballots*, specifying which alternatives are “approved” by the voters. For an overview of research on approval-based committee elections, we refer to the recent survey by Lackner and Skowron [31].

A crucial issue in group decision making is *(proportional) representation*. Informally speaking, an outcome of a decision-making process is representative if it reflects the preferences of the members of the group. In the context of approval-based committee elections, reasoning about representation is non-trivial. Since approval sets may overlap arbitrarily, there are many different ways in which the set of voters can be split into more or less “cohesive” subgroups. Whether a given subgroup has a justified claim to be represented in the committee depends on the size of the subgroup as well as on its level of cohesiveness.

Aziz et al. [5] and Sánchez-Fernández et al. [56] have identified axiomatic properties capturing the intuitive notion that subgroups that are “large enough” and “cohesive enough” deserve to be represented in the committee: *justified representation (JR)*, *proportional justified representation (PJR)*, and *extended justified representation (EJR)*. While a number of standard committee voting rules have been shown to satisfy the basic requirement of JR, it turns out that the more demanding properties PJR and EJR are much harder to satisfy.

In this paper, we consider committee voting rules that are due to Swedish mathematician Edvard Phragmén (we provide brief biographical information in Sect. 1.2). Phragmén phrases committee elections as load balancing problems: Adding a candidate to the committee incurs some *load*, and this load should be shared among the voters approving this candidate. Phragmén suggests choosing committees in such a way that the corresponding load distributions are as *balanced* as possible, and different ways of measuring balancedness result in different optimization objectives. This approach yields two *optimization* variants, one minimizing the maximum load and one minimizing the variance of loads, and one *sequential* variant, which proceeds by greedily selecting candidates so as to keep the maximum load as small as possible. In addition to the load balancing rules, Phragmén also proposed a rule that adapts the principle behind Single Transferable Vote (STV) to approval ballots.

Although Phragmén ’s methods were proposed in the same era as *Proportional Approval Voting (PAV)*,^1^ they have received hardly any attention until very recently. Since the publication of the conference version of this paper [12] in 2017, Phragmén ’s methods became increasingly central in the analysis of approval-based committee rules.^2^ In politics, variants of both Phragmén ’s methods and PAV have been used in Swedish parliamentary elections (for distribution of seats within parties), and a version of one of Phragmén ’s methods is still part of the election law, although in a minor role [26]. Further, Phragmén ’s sequential method is often used for the selection of “validators” who participate in a blockchain consensus protocol: In the recently introduced *nominated proof-of-stake (NPoS)* mechanism, members of a blockchain community can nominate other members to become validators, and the selection of a representative set of validators plays an important role for the security of the blockchain [15, 18, 49].

### Results and outline of the paper

After briefly reviewing related work in Sect. 2 and introducing some basic notation in Sect. 3, we formally define Phragmén ’s methods in Sect. 4. In Sect. 5, we analyze the computational complexity of Phragmén ’s methods and we provide algorithms for computing them. The algorithms for the optimization variants are based on mixed-integer linear and quadratic programming. In Sect. 6, we consider the representation axioms mentioned above. We show that the sequential variant satisfies PJR, making it one of few committee monotonic methods with this property. Moreover, we show that the optimization variants satisfy *perfect representation (PR)*, a further representation axiom introduced by Sánchez-Fernández et al. [56]. The latter result provides a contrast to PAV, which is known to violate PR. In Sect. 7, we discuss the relation between Phragmén ’s methods and the apportionment problem [8].

### A brief biography of Phragmén

Lars Edvard Phragmén (1863–1937) was a Swedish mathematician, actuary and insurance executive. He began his mathematical university studies in Uppsala in 1882, but transferred in 1883 to Stockholm, where he became a student (and later confidant) of Gösta Mittag-Leffler [63]. In 1888, Phragmén was appointed coeditor of Mittag-Leffler’s journal *Acta Mathematica*, where he immediately made an important contribution by finding an error in a paper by Henri Poincaré on the three-body problem. The paper had been awarded a prize in a competition that Mittag-Leffler had persuaded King Oscar II to arrange, but Phragmén found a serious mistake when the journal had already been printed; the copies that had been released were recalled and a new corrected version was printed.

**Fig. 1:**
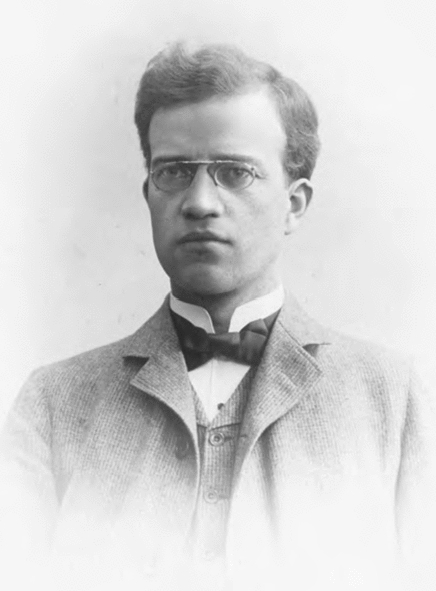
Edvard Phragmén

In 1892, Phragmén became a professor of mathematics at Stockholm University. In 1897, he additionally became an actuary in a private insurance company. His interest in actuarial science and insurance companies appears to have grown in these years, as in 1904 he left his professorship to become the first head of the Swedish Insurance Supervisory Authority. In 1908 he became director of a private insurance company, which he remained until 1933. His involvement in mathematics is witnessed, e.g., by his attendance at the 1924 International Mathematical Congress in Toronto, where he was elected one of the vice-presidents of the International Mathematical Union [16]. Phragmén also continued to be an editor of Acta Mathematica until his death in 1937.

His best known mathematical work is the Phragmén–Lindelöf principle in complex analysis, a joint work with Finnish mathematician Ernst Lindelöf [47]. His interest in election methods is witnessed by his publications [42–46]. Moreover, he was a member of the *Royal Commission on a Proportional Election Method 1902–1903* and of a new *Royal Commission on the Proportional Election Method 1912–1913*. For further information we refer the reader to the survey by Janson [26] and to the book by Stubhaug [63] (in particular for his relation with Mittag-Leffler).

## Related work

Proportional representation is an important issue in committee voting (see the influential paper by Monroe [34] and the references therein) and methods ensuring representation often lead to interesting computational problems [9, 32, 50, 51].

The problem of choosing representative committees based on approval ballots can be seen as a generalization of the classical *apportionment* problem [8]. The latter setting corresponds to the special case in which candidates are arranged into party lists and each voter chooses a single list; see Sect. 7 for details. Voting settings between apportionment and approval-based committee voting have also been studied [14].

For the setting of approval-based committee voting [29, 31], Aziz et al. [5] proposed two representation axioms: *justified representation (JR)* and its strengthening *extended justified representation (EJR)*. Later, Sánchez-Fernández et al. [56] observed that EJR is not compatible with what they call *perfect representation* and proposed an axiomatic property, *proportional justified representation (PJR)*, that is compatible. EJR implies PJR, which in turn implies JR.

Aziz et al. [5] and Sánchez-Fernández et al. [56] showed that most common committee voting rules fail EJR and PJR. A notable exception is Thiele’s PAV [64], which satisfies EJR (and thus PJR). Interestingly, variants of PAV based on different weight vectors fail both EJR and PJR (and even weaker proportionality requirements) [5, 13]. Moreover, a greedy approximation algorithm for PAV known as *sequential PAV* or *reweighted approval voting* fails JR (and consequently PJR and EJR) [5, 56].

Computing the outcome of PAV is NP-hard [4, 60] and thus not feasible in polynomial time unless $\text {P}=\text {NP}$. Prior to our work, it had remained an open question whether there exist polynomial-time computable rules satisfying EJR or PJR. Phragmén ’s sequential rule, as we show in this paper, is polynomial-time computable and satisfies PJR.

Recent work has established that even EJR can be guaranteed by a polynomial-time voting rule. This was first shown by Aziz et al. [6]. Later, Peters and Skowron [40] presented the Method of Equal Shares (MES), which is also polynomial-time computable and satisfies EJR. Interestingly, MES is based on the same principle as Phragmén ’s sequential method and shares some of its desirable properties (such as laminar proportionality and priceability [40]). None of these rules, however, are committee monotonic,^3^ i.e., an increase in the committee size by one may result in a completely different committee. In many settings, committee monotonicity is highly desirable (e.g., when generating rankings [24, 53, 61]), and thus Phragmén ’s sequential method—which is committee monotonic by definition—has gained much attention in recent years. Phragmén ’s sequential method also satisfies further monotonicity axioms [26, 54].

The maximin support method, introduced by Sánchez-Fernández et al. [57], is closely related to Phragmén ’s sequential method and shares many of its axiomatic properties (including PJR and committee monotonicity). The optimization variant of the maximin support method coincides with one of the optimization variants of Phragmén ’s methods, and yields an equivalent formulation of the latter in terms of maximin support [57]. An interesting distinction between Phragmén ’s sequential rule and the maximin support method concerns their ability to approximate the optimal solution of the maximin support problem [18].

Proportional representation has also been studied in settings where voters have ordinal preferences over candidates [19, 21] and in *participatory budgeting*, a generalization of committee elections where candidates have costs and the set of selected candidates needs to satisfy a budget constraint [3, 41]. Different variants of Phragmén ’s methods have been generalized to those settings [1, 7, 26]. Further generalizations of Phragmén ’s methods have been considered in the context of degressive and regressive proportionality [28] and in the context of perpetual voting [30].

## Preliminaries

We consider a social choice setting with a finite set $N=\{1,\ldots , n\}$ of *voters* and a finite set *C* of *candidates*. Throughout the paper we let $m = |C|$ denote the number of candidates and $n=|N|$ the number of voters. The preferences of each voter $i\in N$ are given by a subset $A_i\subseteq C$, representing the subset of candidates that the voter approves of. We refer to the list $A = (A_1,\ldots , A_n)$ as the *preference profile*. For a candidate $c \in C$, we let $N_c$ denote the set of voters approving *c*, i.e., $N_c = \{i\in N \mathrel {:}c \in A_i\}$. To avoid trivialities, we assume that $N_c\ne \emptyset$ for all $c \in C$.

We want to select a subset consisting of exactly *k* candidates, for a given natural number $k \le m$. An *approval-based committee voting rule* (henceforth simply *rule*) maps an instance (*A*, *k*) to a subset $S \subseteq C$ of size *k*, the *committee*. In general, there may be ties, and we then allow the rule to yield several choices, so formally the rule is a map from instances to non-empty sets of committees.

Finally, for a tuple of real numbers $z=(z_1,\dots ,z_n)$, we let $z_{(\ell )}$ denote the $\ell$-th largest element in *z*, so that $z_{(1)} \ge z_{(2)} \ge \dots \ge z_{(n)}$.

## Phragmén ’s methods

The main idea behind Phragmén ’s methods is to identify committees whose “support” is distributed as evenly as possible among the electorate. Phragmén used different formulations for explaining his methods; we refer the reader to the survey by Janson [26] for an overview and more details. In this paper, we adopt the formulation from the 1899 paper [46]. In this formulation, every candidate in the committee is thought of as incurring one unit of “*load*,” and the load incurred by candidate *c* needs to be distributed among the voters in $N_c$. The goal is to find a committee of size *k* for which the corresponding load distribution is as balanced as possible.

Formally, a *load distribution* is a two-dimensional array $x = (x_{i,c})_{i \in N, c \in C}$ satisfying the following four constraints:

4.1 $$\begin{aligned}&0 \le x_{i,c} \le 1&\text { for all }i \in N \text { and }c \in C \end{aligned}$$

4.2 $$\begin{aligned}&x_{i,c} = 0&\text { if }c\notin A_i \end{aligned}$$

4.3 $$\begin{aligned}&\sum _{i\in N} \sum _{c\in C} x_{i,c} = k&\end{aligned}$$

4.4 $$\begin{aligned}&\sum _{i\in N} x_{i,c} \in \{0,1\}&\text { for all }c\in C \end{aligned}$$

Here, $x_{i,c}$ corresponds to the load that voter *i* receives from candidate *c*. Constraint (4.2) ensures that the load incurred by candidate *c* is distributed among voters in $N_c$ only, and constraints (4.3) and (4.4) ensure that *x* corresponds to a size-*k* committee $\{c \in C \mathrel {:}\sum _{i \in N} x_{i,c} = 1\}$.

For a load distribution *x*, we let ${\bar{x}}_i$ denote the total load of voter $i \in N$, i.e., ${\bar{x}}_i=\sum _{c\in C} x_{i,c}$, and we refer to $({\bar{x}}_1, \ldots , {\bar{x}}_n)$ as the vector of *voter loads*. Using this notation, constraint (4.3) reads $\sum _{i\in N} {\bar{x}}_i = k$. Note that constraint (4.3) implies that the *average* voter load is $\frac{k}{n}$.

There are different ways of measuring how *balanced* a given load distribution is, each giving rise to a different optimization objective. One such objective is to minimize the maximum load assigned to a voter, i.e., $\min _x \max _{i\in N}{\bar{x}}_i$. (This is equivalent to minimizing the maximum difference between a voter load and the average voter load.) Obviously, the average voter load $\frac{k}{n}$ is a lower bound on the maximum voter load, and we call a load distribution *x* *perfect* if ${\bar{x}}_i = \frac{k}{n}$ for all $i \in N$. Another objective is to minimize the *variance* of voter loads, i.e., the sum of squared distances from the average voter load. Again, a perfect load distribution is optimal for this objective.

We further distinguish between “optimization ” methods, where we solve a global optimization problem to find a load distribution optimizing the objective, and “sequential” methods, where we iteratively construct a load distribution, in each round greedily choosing a candidate optimizing the objective at that iteration.

In this paper, we focus on three rules: the optimization methods *leximax-Phragmén* and *var-Phragmén*—minimizing the maximum voter load and the variance of voter loads, respectively—and the sequential method *seq-Phragmén*, which greedily minimizes the maximum voter load. For completeness, we also consider the *Eneström-Phragmén* method (see Sect. 4.3).

The method seq-Phragmén was introduced by Phragmén in several papers [43–46], and it is the variant that he proposed to be used in actual elections. Phragmén defined this method as a generalization of D’Hondt’s apportionment method to the case without party lists (see Sect. 7). Optimization variants and the objective of minimizing the variance are discussed in the 1896 paper [45].

### Optimization variants

We start by defining the optimization variants. The first optimization variant selects committees corresponding to load distributions minimizing the maximum voter load. In case that two or more committees have the same (minimal) maximum load, we employ a specific way of breaking ties. This is because it might be the case that for two load distributions *x* and *y*, although $\max _{i\in N}{\bar{x}}_i=\max _{i\in N}{\bar{y}}_i$, one load distribution is clearly preferable to the other.

#### Example 4.1

Let $C = \{ a, b, c \}$, $k=2$, and $A=(\{a\},\{a\},\{b\},\{c\})$. Any committee of size 2 contains either *b* or *c*, which are approved by only one voter each, so the maximum load is 1 for all committees. However, the committees containing *a* represent three voters, while the committee $\{ b, c \}$ only represents two.

In order to refine the set of winning committees, we compare two vectors of voter loads according to the *leximax* ordering.^4^

#### Definition 4.2

For $y=(y_1,\dots ,y_n)$ and $z=(z_1,\dots ,z_n)$, *y* is *leximax-smaller* than *z*, denoted $y \mathbin {\dot{<}} z$, if there exists $j\le n$ such that $y_{(j)} < z_{(j)}$ and $y_{(i)} = z_{(i)}$ for all $i\le j-1$.

We are now ready to define the first optimization variant.

**leximax-Phragmén:** The rule leximax-Phragmén selects all committees corresponding to load distributions *x* such that $({\bar{x}}_{1},\dots , {\bar{x}}_{n})$ is leximax-optimal, i.e., minimal with respect to $\mathbin {\dot{<}}$.

As we will see in Sect. 6.3, leximax tie-breaking is necessary in order to guarantee strong representation properties.

The second optimization variant is based on a different optimization objective.

**var-Phragmén:** The rule var-Phragmén selects all committees corresponding to load distributions minimizing $\sum _{i\in N} {\bar{x}}_i^{\,2}$.

Minimizing $\sum _{i\in N} {\bar{x}}_i^{\,2}$ indeed minimizes the variance of $({\bar{x}}_1, \dots , {\bar{x}}_n)$, as is well-known: Since $\frac{1}{n} \sum _{i\in N} {\bar{x}}_i = \frac{k}{n}$, it holds that the variance of $({\bar{x}}_1, \dots , {\bar{x}}_n)$ equals

$$\begin{aligned} \frac{1}{n}\sum _{i\in N}&\left( {\bar{x}}_i -\frac{k}{n}\right) ^2 = \frac{1}{n}\sum _{i\in N} \left( {\bar{x}}_i^{\, 2}-2 {\bar{x}}_i\cdot \frac{k}{n} + \frac{k^2}{n^2}\right) \\&= \frac{1}{n}\sum _{i\in N} {\bar{x}}_i^{\, 2} - \frac{1}{n} \cdot 2 k\cdot \frac{k}{n} + \frac{1}{n}\cdot n\cdot \frac{k^2}{n^2} \\&= \frac{1}{n}\sum _{i\in N} {\bar{x}}_i^{\, 2} - \frac{k^2}{n^2}. \end{aligned}$$

When minimizing this expression, we can ignore multiplicative or additive constants (*n* and *k*) and thus equivalently minimize $\sum _{i\in N} {\bar{x}}_i^{\,2}$.

The following example demonstrates that the maximum voter load under var-Phragmén may indeed be greater than under leximax-Phragmén.

#### Example 4.3

**Fig. 2:**
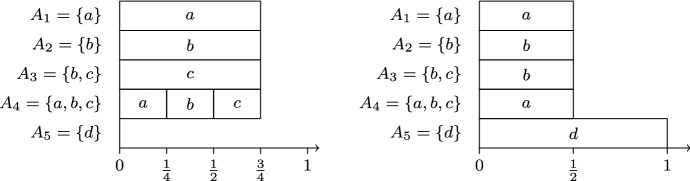
Illustration of Example 4.3. The diagram on the left illustrates a load distribution minimizing the maximum voter load $\max _{i\in N} {\bar{x}}_i$, and the diagram on the right illustrates the unique load distribution minimizing $\sum _{i\in N} {\bar{x}}_i^2$

Let $C = \{a,b,c,d\}$, $k=3$, and consider the preference profile $A = (\{a\} , \{b \}, \{b, c\} , \{a, b, c\} , \{d\})$. For this instance, leximax-Phragmén selects the committee $\{a,b,c\}$ and var-Phragmén selects the committee $\{a,b,d\}$. Optimal load distributions corresponding to these committees are illustrated in Fig. 2. Load distributions minimizing the maximum voter load (like the one illustrated by the first diagram in Fig. 2) satisfy $\max _{i\in N} {\bar{x}}_i = \frac{3}{4}$ and $\sum _{i\in N} {\bar{x}}_i^2 = 4 (\frac{3}{4})^2 = \frac{9}{4}$, and the load distribution minimizing the variance of voter loads (illustrated by the second diagram in Fig. 2) satisfies $\max _{i\in N} {\bar{x}}_i = 1$ and $\sum _{i\in N} {\bar{x}}_i^2 = 4 (\frac{1}{2})^2 + 1^2 = 2$.

#### Remark 4.4

Rather than minimizing the maximum load, one could also aim to *maximize the minimum voter load*. This variant would select committees minimizing the number of unrepresented voters, even in the face of large cohesive groups of voters. Therefore, this method will not do well in terms of the representation axioms considered in Sect. 6. For this reason, we do not consider it further in this paper.

### Sequential method

We now introduce the sequential method, which can be seen as a greedy algorithm for minimizing the maximum voter load.

**seq-Phragmén:** The rule seq-Phragmén starts with an empty committee and iteratively adds candidates, always choosing the candidate that minimizes the (new) maximum voter load (under the assumption that previously assigned loads cannot be redistributed). Let ${\bar{x}}_i^{(j)}$ denote the voter loads after round *j*. At first, all voters have a load of 0, i.e., ${\bar{x}}_i^{(0)}=0$ for all $i\in N$. In each round, we keep the already assigned loads, but we may further increase them and give the additional load to a new candidate *c*. In other words, we require ${\bar{x}}_i^{(j)}\ge {\bar{x}}_i^{(j-1)}$ for all *i*, with equality unless $i\in N_c$. Moreover, the sum of the loads added in the round should be 1. (Hence, the total load after *j* rounds is *j*, which is the sequential version of constraint (4.3).) We select the candidate *c* and the loads ${\bar{x}}_i^{(j)}$ that satisfy these conditions and minimize $\max _i {\bar{x}}_i^{(j)}$. (If there are several candidates achieving the minimum, we use a fixed tie-breaking rule to decide which candidate to add.)

The candidates and loads chosen by this procedure have the following properties.

#### Lemma 4.5

In round *j*, given the voter loads ${\bar{x}}_i^{(j-1)}$ for all $i\in N$ and a candidate *c* that was not selected in earlier rounds, let

4.5 $$\begin{aligned} s_c^{(j)} = \frac{1 + \sum _{i\in N_c} {\bar{x}}_i^{(j-1)}}{|N_c|}. \end{aligned}$$

Then, the maximum load $s^{(j)}=\max _i {\bar{x}}_i^{(j)}$ after round *j* will be

4.6 $$\begin{aligned} s^{(j)}=\min _c s_c^{(j)}, \end{aligned}$$

taking the minimum over the candidates that remain in round *j*, and a candidate *c* is elected that achieves the minimum in (4.6). Moreover, if *c* is elected, the new loads after round *j* will be

4.7 $$\begin{aligned} {\bar{x}}_i^{(j)} = {\left\{ \begin{array}{ll} s_c^{(j)} &{} \text {if } i \in N_c\\ {\bar{x}}_i^{(j-1)} &{} \text {otherwise.} \end{array}\right. } \end{aligned}$$

Furthermore, both individual loads and the maximum load sequence are weakly increasing: $0\le {\bar{x}}_i^{(1)} \le \ldots \le {\bar{x}}_i^{(k)}$ for every $i \in N$, and $0\le s^{(1)} \le \ldots \le s^{(k)}$.

#### Proof

We use induction on *j*, so we assume that the claims hold for all rounds before *j*. We claim first that the following inequalities hold for every remaining candidate *c* and for all $i\in N$:

4.8 $$\begin{aligned} s_c^{(j)} \ge s^{(j-1)} \ge {\bar{x}}_i^{(j-1)}. \end{aligned}$$

It is obvious that (4.8) holds for $j=1$. If $j>1$, then, by the induction hypothesis, ${\bar{x}}_i^{(j-1)}\ge {\bar{x}}_i^{(j-2)}$ for every *i*. Hence, (4.5) yields $s_c^{(j)} \ge s_c^{(j-1)}$ for every remaining candidate *c*. Furthermore, (4.6) (for $j-1$) yields $s_c^{(j-1)} \ge s^{(j-1)}$ for every remaining candidate *c*, and thus (4.8) holds in this case too, recalling the definition $s^{(j-1)}=\max _i {\bar{x}}_i^{(j-1)}$.

Next, since (4.8) holds, for any remaining candidate *c*, the assignment (4.7) satisfies ${\bar{x}}_i^{(j)}\ge {\bar{x}}_i^{(j-1)}$ for every *i*, with equality if $i\notin N_c$. Moreover, the sum of the added loads is, by (4.7) and (4.5),

4.9 $$\begin{aligned} \sum _{i\in N_c}\left( {\bar{x}}_i^{(j)}-{\bar{x}}_i^{(j-1)}\right) =|N_c| s_c^{(j)}-\sum _{i\in N_c}{\bar{x}}_i^{(j-1)} =1. \end{aligned}$$

Thus, (4.7) yields a valid load distribution for round *j*. It follows from (4.8) that its maximum load is $s_c^{(j)}$.

Conversely, any distribution of an additional load 1 on the voters in $N_c$ will give these voters an average load of $s_c^{(j)}$, and thus the maximum load will be at least $s_c^{(j)}$ (and strictly greater for loads differing from (4.7)).

Hence, the maximum load after round *j* is minimized by one of the assignments (4.7), where obviously *c* should be chosen to minimize $s_c^{(j)}$. This proves (4.6), and the remaining assertions follow. $\square$

Note that (4.5)–(4.7) (together with a tie-breaking rule) give a simple polynomial-time algorithm for computing the outcome of seq-Phragmén: In each round *j*, compute $s_c^{(j)}$ for all remaining candidates *c*, select a candidate minimizing this quantity (potentially using the tie-breaking rule), and update voter loads according to (4.7). We analyze the running time of this algorithm in more detail in Sect. 5.

Phragmén [46] illustrates his sequential method by imagining the different ballots as represented by cylindrical vessels, with base area proportional to the number of voters casting that ballot. The already elected candidates are represented by a liquid that is fixed in the vessels, and the additional unit of load incurred by adding another candidate to the committee is represented by pouring 1 unit of a liquid into the vessels representing voters approving this candidate. The liquid then distributes among these vessels so that the height of the liquid is the same in all vessels. This is to be tried for each candidate; the candidate that requires the smallest height is elected, and the corresponding amounts of liquid are added to the vessels and fixed there.

An alternative interpretation of the sequential method is in terms of money: Imagine that voters have initially empty bank accounts and earn money continuously (at a constant rate) over time. As soon as the approvers of a candidate jointly own one dollar, they “buy” this candidate and their bank accounts are reset to zero. This interpretation was utilized by Peters and Skowron [40] when introducing the Method of Equal Shares.

Phragmén ’s sequential method is committee monotonic by definition. As mentioned above, seq-Phragmén can be seen as a (polynomial-time computable) heuristic to approximate the optimization method leximax-Phragmén. Unsurprisingly, the load distribution constructed by seq-Phragmén might not be optimally balanced.^5^

#### Example 4.6

**Fig. 3:**
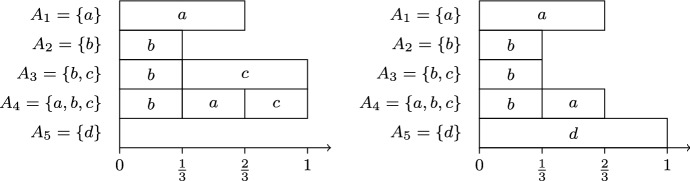
Illustration of Example 4.6. The diagram on the left (respectively, right) illustrates the load distributions obtained by seq-Phragmén with ties broken in favor of candidate *c* (respectively, *d*)

Consider again the instance from Example 4.3. In the first round, we have $s_b^{(1)}=\frac{1}{3}$, $s_a^{(1)}=s_c^{(1)}=\frac{1}{2}$, and $s_d^{(1)}=1$. Therefore, candidate *b* is chosen. In the second round, we have $s_a^{(2)} = \frac{2}{3}$, $s_c^{(2)}=\frac{5}{6}$, and $s_d^{(2)}=1$, so candidate *a* is chosen. In the third round, there is a tie between *c* and *d* because $s_c^{(3)}=s_d^{(3)}=1$. Thus, the final committee is either $\{a,b,c\}$ or $\{a,b,d\}$, depending on which tie-breaking rule is used. Figure 3 illustrates the resulting load distributions, both of which are suboptimal for the optimization problems corresponding to leximax-Phragmén and var-Phragmén.

One can also define a sequential version of var-Phragmén, by in each iteration selecting a candidate minimizing the variance of the resulting load distribution [35]. This variant does not fare well in terms of the representation axioms considered in Sect. 6, and we therefore do not consider it any further.

### Eneström-Phragmén method

In addition to the methods described in the previous sections, there is another rule that is attributed, at least partially, to Phragmén.^6^ Following Camps et al. [17], we refer to this method as *Eneström-Phragmén*.

The method predates the load balancing methods and is similar in spirit to single transferable vote (STV) methods [65]. It uses a quota *q*, which is defined either as the *Hare quota* $q_H = \frac{n}{k}$ or as the *Droop quota* $q_D = \frac{n}{k+1}$. The choice between $q_H$ and $q_D$ does not affect the axiomatic performance of the rule with respect to the properties studied in this paper. While Eneström-Phragmén is indistinguishable from seq-Phragmén with respect to the representation properties studied in Sect. 6, a crucial difference is that Eneström-Phragmén is not committee monotonic [17].

**Eneström-Phragmén:** Initially, all voters have a voting weight of 1. Each ballot is counted fully, with its present voting weight, for each unelected candidate on the ballot. In each round, a candidate with maximum weighted approval score is chosen and the voting weights of voters approving this candidate are reduced: If the maximum weighted approval score *v* is strictly greater than the quota (i.e., $v > q$), then each of these ballots has its voting power multiplied by $\frac{v-q}{v}$; if $v \le q$, then these ballots all get voting power 0 (and are thus ignored in the sequel). This is repeated until the desired number of candidates are elected.

Note that the total voting weight of all voters is decreased by $(q/v) \cdot v = q$ each time, as long as some candidate reaches the quota. This rule has been extensively analyzed by Camps et al. [17] (mostly using $q_D$). Independently, it has been studied by Sánchez-Fernández et al. [55] (using $q_H$). In the following example, we use $q_H$.

#### Example 4.7

Consider again the instance from Example 4.3. We have $q_H=\frac{n}{k}=\frac{5}{3}$. In the first round, candidate *b* is chosen with a (weighted) approval score of 3. Since $3>q_H$, the voting power of the three voters approving *b* is multiplied by $\frac{3-q_H}{3}=\frac{4}{9}$. In the second round, the weighted approval scores of the remaining candidates are $1+\frac{4}{9} = \frac{13}{9}$ for *a*, $\frac{4}{9} +\frac{4}{9} = \frac{8}{9}$ for *c*, and 1 for *d*. Therefore, candidate *a* is chosen. Since $\frac{13}{9} \le q_H$, both voters approving *a* have their voting power reduced to 0. In the third and final round, the weighted approval score of *c* is $\frac{4}{9}$ and candidate *d* is chosen with a weighted approval score of 1.

## Computational aspects

In this section, we study the computational complexity of Phragmén ’s methods, and we provide algorithms for finding winning committees. Sánchez-Fernández et al. [56] have shown that every rule satisfying *perfect representation* (see Sect. 6) is NP-hard to compute; this essentially follows from earlier work by Procaccia et al. [51]. Since we show that leximax-Phragmén and var-Phragmén both satisfy this condition (Theorems 6.10 and 6.14), it follows that there do not exist polynomial-time algorithms for computing a committee for either of these rules, unless $\text {P}=\text {NP}$.

We complement these hardness results by considering two basic decision problems. leximax-Phragmén asks whether an instance allows a load distribution *x* such that $({\bar{x}}_{1},\dots , {\bar{x}}_{n}) \mathbin {\dot{<}} (y_1,\dots ,y_n)$ for some given *n*-tuple $(y_1,\dots ,y_n) \in \mathbb {R}_{\ge 0}^n$. var-Phragmén asks whether an instance allows a load distribution *x* such that $\sum _{i\in N} {\bar{x}}_{i}^{\,2} < \alpha$ for some given threshold value $\alpha > 0$. Both problems can be interpreted as asking whether a given load distribution is optimal. We show that both problems are NP-complete even for rather restricted instances. For a preference profile *A*, let *s*(*A*) denote the maximum number of candidates a voter approves, and let *d*(*A*) denote the maximum number of voters that approve a candidate.

### Theorem 5.1

The decision problems leximax-Phragmén and var-Phragmén are NP-complete, even restricted to instances with $s(A)=2$ and $d(A)=3$.

### Proof

To show hardness for both problems, we reduce from the NP-complete problem Independent Set on cubic graphs [22, 23], which is defined as follows: given a cubic graph (*V*, *E*) (i.e., a graph such that every vertex has degree 3) and a positive integer *k*, is there a set of vertices $S\subseteq V$ with $|S|=k$ such that $|e\cap S|\le 1$ for all edges $e\in E$? Let $E=(e_1,\dots ,e_n)$. We construct an instance of leximax-Phragmén and var-Phragmén by identifying candidates with vertices ($C=V$) and voters with edges, i.e., $A=(e_1,\dots ,e_n)$. It is easy to see that $s(A)=2$ and $d(A)=3$. Without loss of generality we assume that $n\ge 3k$ because cubic graphs with fewer than 3*k* edges cannot have an independent set of size *k*.^7^

To prove that leximax-Phragmén is NP-hard, we claim that (*V*, *E*) has an independent set of size *k* if and only if there exists a load distribution *x* with $({\bar{x}}_{1},\dots , {\bar{x}}_{n}) \mathbin {\dot{<}} (y_1,\dots ,y_n)$, where $(y_1,\dots ,y_n)$ is the sequence containing 3*k* entries of $\frac{1}{3} + \frac{1}{9k}$ followed by zeros. If *S* is an independent set, then *S*, viewed as a committee, contains candidates that are approved by disjoint sets of (three) voters. Hence, there are exactly 3*k* voters that bear a load of $\frac{1}{3}$; all others have load 0. Conversely, let *S* be a committee such that $({\bar{x}}_{1},\dots , {\bar{x}}_{n}) \mathbin {\dot{<}} (y_1,\dots ,y_n)$. Since candidates are approved by three voters, if there exists a voter with more than one approved candidate in *S*, then the average load (and thus the maximum load) is at least $\frac{k}{3k-1}>\frac{1}{3}+\frac{1}{9k}$, which contradicts our assumption that $({\bar{x}}_{1},\dots , {\bar{x}}_{n}) \mathbin {\dot{<}} (y_1,\dots ,y_n)$. Hence *S* is an independent set.

To prove that var-Phragmén is NP-hard, we claim that (*V*, *E*) has an independent set of size *k* if and only if there exists a load distribution *x* with $\sum _{i\in N} {\bar{x}}_{i}^{\,2}< \frac{k}{3} + \frac{1}{9}$. It is straightforward to see that an independent set *S* corresponds to a committee with $\sum _{i\in N} {\bar{x}}_{i}^{\,2}= 3k\cdot \left( \frac{1}{3}\right) ^2=\frac{k}{3}$. For the other direction, let *S* be a committee with $\sum _{i\in N} \bar{x}_{i}^{\,2}< \frac{k}{3}+\frac{1}{9}$. Note that at most 3*k* voters have approved candidates in the committee. Let $N'\subseteq N$ be such that it contains all voters *i* with ${\bar{x}}_i>0$. Hence $\sum _{i\in N} \bar{x}_{i}^{\,2}=\sum _{i\in N'} {\bar{x}}_{i}^{\,2}$. The value of $\sum _{i\in N'} {\bar{x}}_{i}^{\,2}$ is minimal only if all ${\bar{x}}_i$, $i\in N'$, are equal and we then have $\sum _{i\in N'} {\bar{x}}_{i}^{\,2} = |N'|\cdot (\frac{k}{|N'|})^2 =\frac{k^2}{|N'|}$. If $|N'|<3k$, we thus see that $\sum _{i\in N'} {\bar{x}}_{i}^{\,2} \ge \frac{k^2}{3k-1} > \frac{k}{3}+\frac{1}{9}$. Hence $|N'|=3k$ and we can conclude that *S* corresponds to an independent set.

It remains to be shown that leximax-Phragmén and var-Phragmén are contained in NP. This is not immediate as a witness for a Yes-Instance (i.e., a load distribution) may not have a polynomially-sized bit representation. In other words, the fractions in the load distribution may have very large numerators and denominators. To resolve this issue, we encode leximax-Phragmén as a mixed-integer linear program (see the discussion following this proof). Solving a mixed-integer linear program (i.e., its corresponding decision problem) is known to be NP-complete [59].^8^ For showing NP-membership of var-Phragmén, we proceed in a similar fashion: we encode it as a mixed-integer quadratic program (see Theorem 5.3). NP-membership then follows from a result by Pia et al. [48]. $\square$

We now turn to algorithms for computing Phragmén ’s methods. First, we show how the outcome of leximax-Phragmén can be computed with the help of mixed-integer linear programs (MILPs).^9^ We start by formulating a MILP that solves the decision problem leximax-Phragmén. We are thus given a load vector $\textbf{y} = (y_1, \ldots , y_n)$ and ask whether an improvement is possible. Without loss of generality we assume that $y_1 \ge \ldots \ge y_n$. The general idea is to find an index *t* where an improvement over $\textbf{y} = (y_1, \ldots , y_n)$ is possible. This requires a new load vector $\textbf{x} = ({\bar{x}}_1, \ldots , {\bar{x}}_n)$ such that ${\bar{x}}_{(1)},\dots ,{\bar{x}}_{(t-1)}$ remain equal to $y_1, \ldots , y_{t-1}$, respectively, and that ${\bar{x}}_{(t)},\dots ,{\bar{x}}_{(n)}$ are each less than or equal to $y_t - \epsilon$ for some $\epsilon >0$. We thus guess the index *t* and a mapping from indices $1,\dots ,t-1$ to voters.

We use variables $x_{i,c}$ (for $i\in N$, $c\in C$), $e_{i,j}$ (for $i,j\in N$), $s_{i}$ (for $i\in N$), $t_{j}$ (for $j\in N$), and $\epsilon$. Recall that ${\bar{x}}_i=\sum _{c\in C} x_{i,c}$. For a given *n*-tuple $\textbf{y}$, let $\textsf {P}(\textbf{y})$ be the MILP that maximizes $\epsilon$ under the constraints (4.1)–(4.4) and (5.1)–(5.8).

5.1 $$\begin{aligned}&e_{i,j} \in \{0,1\}&\quad \text { for all }i,j \in N \end{aligned}$$

5.2 $$\begin{aligned}&s_i \in \{0,1\}&\text { for all }i \in N \end{aligned}$$

5.3 $$\begin{aligned}&t_j \in \{0,1\}&\text { for all }j \in N \end{aligned}$$

5.4 $$\begin{aligned}&s_i+\sum _{j \in N} e_{i,j} = 1&\text { for all }i \in N \end{aligned}$$

5.5 $$\begin{aligned}&t_j+\sum _{i \in N} e_{i,j} \le 1&\text { for all }j \in N \end{aligned}$$

5.6 $$\begin{aligned}&\sum _{j \in N} t_j = 1&\end{aligned}$$

5.7 $$\begin{aligned}&{\bar{x}}_i - k (1 - e_{i,j}) \le y_j&\text { for all }i,j \in N \end{aligned}$$

5.8 $$\begin{aligned}&{\bar{x}}_i - k (2 - s_i - t_j) \le y_j -\epsilon&\text { for all }i,j \in N \end{aligned}$$

This MILP can be understood as follows: The variables $e_{i,j}$ encode a partial bijection $\pi$ from a subset of *N* to a subset of *N* (those indices where no improvement occurs); the variables $s_i$ encode the subset $S \subseteq N$ where $\pi$ is not defined (those indices where the loads are less than or equal to $y_t-\epsilon$); and the variables $t_j$ encode $t\in N$, an index of an element in $\{y_j: j\notin \textit{range}(\pi )\}$ (the index *t* where an actual improvement occurs). Constraint (5.4) encodes the relation between $\pi$ and *S*: for every $i\in N$, either $s_i=1$ or $e_{i,j}=1$ for some $j\in N$. In a similar fashion, constraint (5.5) encodes the relation between $\pi$ and *t*: for every $i\in N$, $t_i=1$ only if $e_{i,j}=0$ for all $j\in N$. Together with constraint (5.6), we enforce that there exists exactly one $j\in N$ such that $t_j=1$. Hence at least one voter has a load strictly smaller than $y_t$ and $({\bar{x}}_{1},\dots , {\bar{x}}_{n}) \mathbin {\dot{<}} (y_1,\dots ,y_n)$.

The final two constraints ensure that indeed $({\bar{x}}_{1},\dots , {\bar{x}}_{n}) \mathbin {\dot{<}} (y_1,\dots ,y_n)$. From constraint (5.7) it follows that ${\bar{x}}_i \le y_j$ whenever $\pi (i)=j$. This is because if $e_{i,j}=0$ (i.e., $\pi (i)\ne j$), constraint (5.7) reduces to ${\bar{x}}_i - k \le y_j$, which is trivially satisfied because every load distribution *x* satisfies ${\bar{x}}_i \le k$ for all $i \in N$. If $e_{i,j}=1$ (i.e., $\pi (i)= j$), however, constraint (5.7) reads ${\bar{x}}_i \le y_j$. Similarly, constraint (5.8) enforces that ${\bar{x}}_i\le y_t -\epsilon \le \max _{j\in N\setminus \textit{range}(\pi )} y_j -\epsilon$ for $i\in S$. As we maximize $\epsilon$, we look for a solution where ${\bar{x}}_i < \max _{j\in N\setminus \textit{range}(\pi )} y_j$. We conclude that a feasible solution with objective function value $\epsilon >0$ encodes a load distribution *x* with $({\bar{x}}_{1},\dots , {\bar{x}}_{n}) \mathbin {\dot{<}}(y_1,\dots ,y_n)$. Observe that $\textsf {P}(\textbf{y})$ solves the leximax-Phragmén decision problem: given voter loads $\textbf{y}$, $\textsf {P}(\textbf{y})$ returns $\epsilon >0$ if and only if leximax-Phragmén with input $\textbf{y}$ is a Yes-instance.

We now present a MILP-based algorithm that computes the outcome of leximax-Phragmén. Our algorithm solves a sequence of at most 2*n* instantiations of the MILP $\textsf {P}$, using the optimal solutions of previously solved instances as constraints for subsequent calls. We assume that $\textsf {P}$ returns the load distribution *x* and the objective function value $\epsilon$. For an overview of the procedure, see Algorithm 1.
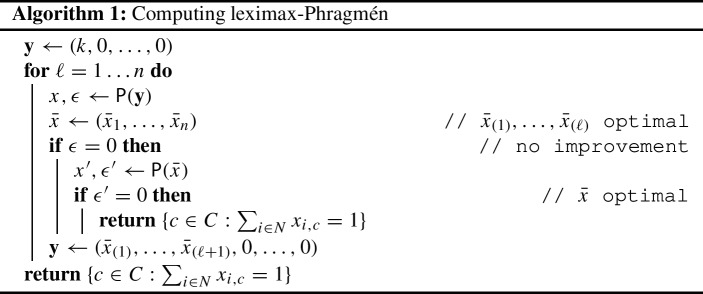


We start with $\textbf{y}=(k,0,\dots ,0)$, an *n*-tuple consisting of one *k* and $n-1$ zeros. We employ $\textsf {P}$ to find a strictly better solution. The only entry of $\textbf{y}$ that can be improved is $\textbf{y}_{(1)}=k$ and hence the solution *x* returned by $\textsf {P}$ minimizes the largest load; let $\bar{x}_{(1)}$ be the largest load and $\bar{x}_{(2)}$ the second-largest. We repeat this procedure with $\textbf{y}=(\bar{x}_{(1)},\bar{x}_{(2)},0,\dots ,0)$. We already know that $\bar{x}_{(1)}$ is optimal and cannot be further decreased (and 0 cannot be improved), hence the next $\textsf {P}$ instance minimizes the second-largest load. We iterate this process and in step $\ell$ guarantee that the $\ell$-th largest load is optimal. If at some point $\textsf {P}$ returns $\epsilon =0$, we verify whether the current solution is optimal: if $\textsf {P}({{\bar{x}}})$ also returns $\epsilon =0$, the load distribution *x* is indeed optimal and the algorithm terminates. In any case Algorithm 1 returns $\{c \in C \mathrel {:}\sum _{i \in N} x_{i,c} = 1\}$, the committee corresponding to the load distribution *x*.

We have therefore proven the following result.

### Theorem 5.2

leximax-Phragmén can be computed by solving at most 2*n* mixed-integer linear programs with $\mathcal {O}(nm+n^2)$ variables.

To compute var-Phragmén, we solve a mixed-integer quadratic program (MIQP), i.e., a program consisting of linear constraints and a quadratic optimization statement.

### Theorem 5.3

var-Phragmén can be computed by solving one mixed-integer quadratic program with $\mathcal {O}(n m)$ variables.

### Proof

Our MIQP uses the variables $x_{i,c}$ (for $i\in N$, $c\in C$) and the constraints (4.1)–(4.4). The quadratic optimization statement is

$$\begin{aligned} \min \sum _{i\in N} \left( \sum _{c\in C} x_{i,c}\right) ^2. \end{aligned}$$

Since minimizing $\sum _{i\in N} {\bar{x}}_i^{\,2}$ minimizes the variance (see Sect. 4.1), this MIQP computes load distributions corresponding to var-Phragmén committees. $\square$

Finally, we study the runtime for computing seq-Phragmén. A naive estimate is that seq-Phragmén can be computed in ${\mathcal {O}}(kmn)$ time. This estimate ignores the cost of computing the quantities $s_c^{(j)}$, i.e., numerical operations are assumed to require constant time. While this is a sensible assumption in many cases, here it is questionable since computing $s_c^{(j)}$ exactly requires fractions with large numerators and denominators. Indeed, the denominator of $s_c^{(j)}$ can grow exponentially with *j*. Hence, the following theorem also takes the complexity of these operations into account.

### Theorem 5.4

The output of seq-Phragmén can be computed in $\mathcal {O}(k^3mn(\log n)^2)$ time.

### Proof

In the following analysis we also consider the complexity of arithmetic operations in the algorithms, as exact numerical computation of the involved quantities may require numbers of substantial size. Let us consider the procedure described in Sect. 4.2. In each of the *k* rounds, one candidate is chosen. For this, the quantity $s_c^{(j)}$ is computed for every *c* not yet placed in the committee. To ensure correct results, we represent $s_c^{(j)}$ as fractions, i.e., pairs of integers. Let $\{c_1,\dots ,c_{j-1}\}$ be the first $j-1$ chosen candidates. It is easy to see that the denominator of $s_c^{(j)}$ can be bounded by $|N_{c_1}|\cdot \ldots \cdot |N_{c_{j-1}}|\cdot |N_c|\le n^j\le n^k$, assuming we reduce fractions. Furthermore, since $s_c^{(j)}\le k$, the numerator of $s_c^{(j)}$ is at most $kn^k$. Hence, the space required to store $s_c^{(j)}$ is bounded by $\mathcal {O}(k\log n)$. The necessary computations for calculating $s_c^{(j)}$ (addition, division, reducing fractions) can all be performed in ${\mathcal {O}}(b^2)$ time,^10^ where *b* is the number of bits required to store any of $s_c^{(j-1)}$, and $\mathcal {O}(n)$ such operations are required. Since $b= \mathcal {O}(k\log n)$, we conclude that $s_c^{(j)}$ can be computed in $\mathcal {O}(nk^2(\log n)^2)$ time. This has to be done in each of the *k* rounds for at most $|C|=m$ many candidates $c\in C$. The consequent update of ${\bar{x}}_i^{(j)}$ does not increase the runtime bound further. $\square$

## Phragmén ’s methods and representation

In this section, we study which representation axioms are satisfied by Phragmén ’s methods. Our results are summarized in Table 1. Particularly noteworthy are the results that seq-Phragmén satisfies PJR and that leximax-Phragmén and var-Phragmén satisfy PR. For completeness, the table also contains results obtained by Sánchez-Fernández et al. [55] and Camps et al. [17] regarding Eneström-Phragmén.

**Table 1:** Phragmén ’s methods and representation axioms

	JR	PJR	EJR	PR
seq-Phragmén	$\checkmark$ (Corollary 6.8)	$\checkmark$ (Theorem 6.5)	– (Example 6.9)	– (Example 6.3)
leximax-Phragmén	$\checkmark$ (Corollary 6.13)	$\checkmark$ (Theorem 6.11)	– (Example 6.3)	$\checkmark$ (Theorem 6.10)
var-Phragmén	$\checkmark$ (Theorem 6.16)	– (Example 6.15)	– (Example 6.3)	$\checkmark$ (Theorem 6.14)
Eneström-Phragmén	$\checkmark$ [17, 55]	$\checkmark$ [17, 55]	– [17, 55]	– (Example 6.3)

### Representation axioms

We start by stating the definitions of Aziz et al. [5] and Sánchez-Fernández et al. [56].

#### Definition 6.1

A committee $S \subseteq C$ with $|S|=k$ provides

- *justified representation (JR)* if there does not exist a set $N^* \subseteq N$ of voters with $|N^*| \ge \frac{n}{k}$, $|\bigcap _{i \in N^*} A_i| \ge 1$ and $|S \cap A_i| = 0$ for all $i \in N^*$.
- *proportional justified representation (PJR)* if there does not exist an integer $\ell >0$ and a set $N^* \subseteq N$ of voters with $|N^*| \ge \ell \frac{n}{k}$, $|\bigcap _{i \in N^*} A_i| \ge \ell$ and $|S \cap (\bigcup _{i \in N^*} A_i)| < \ell$.
- *extended justified representation (EJR)* if there does not exist an integer $\ell >0$ and a set $N^* \subseteq N$ of voters with $|N^*| \ge \ell \frac{n}{k}$, $|\bigcap _{i \in N^*} A_i| \ge \ell$ and $|S \cap A_i| < \ell$ for all $i \in N^*$.

A rule *f* *satisfies* JR (respectively, PJR or EJR) if, for every instance (*A*, *k*), every committee $S \in f(A, k)$ provides JR (respectively, PJR or EJR).

It follows immediately from the definitions that a rule satisfying EJR also satisfies PJR, and that a rule satisfying PJR also satisfies JR.^11^

The following definition is due to Sánchez-Fernández et al. [56].

#### Definition 6.2

Consider an instance (*A*, *k*) such that *k* divides $$n=|N|$$. A committee $$S = \{c_1, \ldots , c_k\} \subseteq C$$ provides *perfect representation* if there exists a partition of the set *N* of voters into *k* pairwise disjoint subsets $$N_1, \ldots , N_k$$ such that, for all $$j \in \{1,\ldots ,k\}$$, $$|N_j| = \frac{n}{k}$$ and $$c_j \in \bigcap _{i \in N_j} A_i$$. Let $$ PR (A, k)$$ denote the set of all committees providing perfect representation for the instance (*A*, *k*). A rule *f* satisfies *perfect representation (PR)* if, for every instance (*A*, *k*) where *k* divides *n* and $$ PR (A, k) \ne \emptyset $$, we have $$f(A,k) \subseteq PR (A,k)$$.

The following example, which also appears in the papers by Aziz et al. [5] and Sánchez-Fernández et al. [56], illustrates the requirements of the different axioms.

#### Example 6.3

Let $C= \{a,b,c,d,e,f\}$ and consider the 8-voter preference profile given by $A_1=\{a\}$, $A_2=\{b\}$, $A_3=\{c\}$, $A_4=\{d\}$, $A_5=\{a,e,f\}$, $A_6=\{b,e,f\}$, $A_7=\{c,e,f\}$, $A_8=\{d,e,f\}$. Let $k=4$ and assume that ties are broken alphabetically. Then, seq-Phragmén chooses *e*, *f*, *a*, and *b* (in this order). The final loads are $({\bar{x}}_1, \ldots , {\bar{x}}_8) = (\frac{3}{4},\frac{3}{4},0,0,\frac{3}{4},\frac{3}{4},\frac{1}{2},\frac{1}{2})$. This is indeed not optimal as there is a perfect load distribution *y* with ${\bar{y}}_i = \frac{1}{2}$ for all $i \in N$. The corresponding committee $\{a,b,c,d\}$ is selected by both leximax-Phragmén and var-Phragmén.

Let $\ell =2$ and consider the voter group $N^* = \{5, 6, 7, 8\}$ of size $\ell \frac{n}{k} = 2 \frac{8}{4}=4$. Since the voters in $N^*$ all approve candidates *e* and *f*, a set of size $\ell = 2$, the conditions for JR, PJR, and EJR all bind. JR requires that at least one candidate approved by at least one voter in $N^*$ is chosen. PJR requires that at least 2 candidates are chosen that are each supported by at least one voter from $N^*$, while EJR requires that some voter from $N^*$ is represented twice. Thus, EJR dictates that either *e* or *f* is chosen. On the other hand, the only committee providing PR is $\{a,b,c,d\}$. As a consequence, no rule can satisfy both PR and EJR.^12^ Note that leximax-Phragmén and var-Phragmén both violate EJR in this example, and that seq-Phragmén violates PR. Eneström-Phragmén also yields $\{e,f,a,b\}$, and thus violates PR.

### Results for seq-Phragmén

In this section we establish our main result: seq-Phragmén satisfies proportional justified representation.

We use the following notation. For the committee *S* that is selected by seq-Phragmén (using a fixed tie-breaking rule), we can relabel the candidates so that $S=\{c_1 \ldots , c_k\}$ and candidate $c_j$ was chosen in round *j*. Then, we have $c_j = \arg \min _{c \in C \setminus \{c_1, \ldots , c_{j-1}\}} s_c^{(j)}$, and the maximum load after round *j* is $s^{(j)} = s_{c_j}^{(j)}$. The following lemma formalizes the intuitively obvious fact that, when computing the optimal distribution of the load of a candidate *c* among its voters, it never helps to restrict attention to a subset ${N' \subset N_c}$.

#### Lemma 6.4

Fix an instance (*A*, *k*). For $j \le k$, a candidate $c \in C$ that has not been elected before round *j*, and a nonempty subset $N' \subseteq N_c$, let, as a generalization of (4.5),

6.1 $$\begin{aligned} s_c^{(j)}[N'] = \frac{1 + \sum _{i\in N'} {\bar{x}}_i^{(j-1)}}{|N'|}. \end{aligned}$$

Then $s_c^{(j)}[N']$ is the maximum voter load after optimally distributing an additional load of 1 among all voters in $N'$, on top of the loads $\bar{x}_i^{(j-1)}$. In particular, $s_c^{(j)} = s_c^{(j)}[N_c] \le s_c^{(j)}[N']$ for all $N'\subseteq N_c$.

#### Proof

That $s_c^{(j)}[N']$ is the maximum voter load after optimally distributing an additional load 1 among $N'$ follows by Lemma 4.5 (or its proof) by replacing $N_c$ by $N'$; the only non-obvious part is that $s_c^{(j)}[N']\ge {\bar{x}}_i^{(j-1)}$ for all $i\in N'$.

Since the optimal distribution of the addional load among $N'$ is a possible distribution among the larger set $N_c$, it is obvious that the optimal distribution among $N_c$ is at least as good, and thus $s_c^{(j)}[N_c] \le s_c^{(j)}[N']$. $\square$

We are now ready to prove our main theorem.

#### Theorem 6.5

seq-Phragmén satisfies PJR.

#### Proof

PJR requires that $|S \cap (\bigcup _{i \in N^*} A_i)| \ge \ell$ for all groups $N^* \subseteq N$ of voters satisfying $|N^*| \ge \ell \frac{n}{k}$ and $|\bigcap _{i \in N^*} A_i| \ge \ell$ for some integer $\ell >0$. We show that seq-Phragmén satisfies a strictly stronger property by weakening the constraint $|N^*| \ge \ell \frac{n}{k}$ to $|N^*| > \ell \frac{n}{k+1}$.

Consider an instance (*A*, *k*) and let *S* be the committee selected by seq-Phragmén. Assume for contradiction that there exists a voter group $N^* \subseteq N$ and an integer $\ell >0$ with $|N^*| > \ell \frac{n}{k+1}$ such that $|\bigcap _{i \in N^*} A_i| \ge \ell$ and $|S \cap (\bigcup _{i \in N^*} A_i)| \le \ell -1$.

Let $c \in (\bigcap _{i \in N^*} A_i) \setminus S$ and consider round *k* (the last round) of the seq-Phragmén procedure. Adding candidate *c* to the committee would have caused a maximum voter load of

6.2 $$\begin{aligned} s_c^{(k)}&= \frac{1 + \sum _{i\in N_c} {\bar{x}}_i^{(k-1)}}{|N_c|} \le \frac{1 + \sum _{i\in N^*} {\bar{x}}_i^{(k-1)}}{|N^*|} \nonumber \\&\le \frac{1+(\ell -1)}{|N^*|} = \frac{\ell }{|N^*|} < \frac{k+1}{n}. \end{aligned}$$

Here, the first inequality follows from Lemma 6.4 (observe that $N^* \subseteq N_c$), the second inequality follows from $|S \cap (\bigcup _{i \in N^*} A_i)| \le \ell -1$, and the strict inequality follows from $|N^*| > \ell \frac{n}{k+1}$.

Let $c_k$ be the candidate that was chosen in round *k*. Since candidate *c* was *not* chosen, we have $c \ne c_k$ and $s_{c_k}^{(k)} \le s_c^{(k)}$. Using Lemma 4.5 and (6.2), we have $s^{(k)} = s_{c_k}^{(k)} \le s_c^{(k)} <\frac{k+1}{n}$. In particular, this implies that at the end of round *k*, every voter $i \in N$ has a load ${\bar{x}}_i^{(k)}$ that is strictly less than $\frac{k+1}{n}$. Summing the loads over all voters, we get

$$\begin{aligned} \sum _{i \in N} {\bar{x}}_i^{(k)}&= \sum _{i \in N^*} {\bar{x}}_i^{(k)} + \sum _{i \in N \setminus N^*} {\bar{x}}_i^{(k)}\\&\le (\ell - 1) + |N \setminus N^*| \cdot s^{(k)}\\&< \ell - 1 + \frac{n}{k+1} (k+1-\ell ) \frac{k+1}{n} = k, \end{aligned}$$

where we have used the fact that $|N \setminus N^*| \le \frac{n}{k+1} (k+1-\ell )$. But $\sum _{i \in N} {\bar{x}}_i^{(k)} < k$ is a contradiction, because the sum of all voter loads (at the end of the seq-Phragmén procedure) must equal *k*. This completes the proof. $\square$

#### Remark 6.6

We note that the proof of Theorem 6.5 shows that seq-Phragmén satisfies a property that is strictly stronger than PJR, because the constraint on the size of the group $N^*$ has been relaxed.^13^

#### Remark 6.7

In fact, in recent work Peters and Skowron [40] have shown that seq-Phragmén satisfies a stronger property that they call priceability. This in turn implies that seq-Phragmén satisfies Inclusion Proportionality for Solid Coalitions (IPSC) [2], a property that lies between priceability and PJR.^14^

An immediate corollary of Theorem 6.5 is that seq-Phragmén satisfies JR.

#### Corollary 6.8

seq-Phragmén satisfies JR.

However, seq-Phragmén violates EJR, as the following example demonstrates.

#### Example 6.9

Let $C = \{a, b, c_1,c_2, \ldots , c_{12}\}$, $k=12$, and consider the following profile with $n=24$ voters:

$$\begin{aligned}&2 \times \{ a,b,c_1\}{} & {} 6 \times \{ c_1, c_2, \ldots , c_{12}\} \\&2 \times \{ a,b,c_2\}{} & {} 5 \times \{ c_2, c_3, \ldots , c_{12}\} \\{} & {} &9 \times \{ c_3, c_4, \ldots , c_{12}\} \end{aligned}$$

seq-Phragmén selects $S = \{ c_1, c_2, \ldots , c_{12}\}$. (For details of the calculation, see Table 2 in the appendix.) To see that *S* does not provide EJR, consider the group $N^*$ consisting of the four voters on the left. We have $|N^*| = 4 = 2 \frac{n}{k}$ and $|\bigcap _{i \in N^*} A_i| = |\{a,b\}| = 2$. Therefore, EJR requires that at least one voter in $N^*$ approves at least 2 candidates in *S*, which is not the case.

Note that seq-Phragmén also fails PR (see Example 6.3). This is not surprising, considering that PR is computationally intractable [56].

### Results for leximax-Phragmén

In Example 6.3, leximax-Phragmén selects the committee providing perfect representation. We now show that leximax-Phragmén satisfies PR in general.

#### Theorem 6.10

leximax-Phragmén satisfies PR.

#### Proof

Consider an instance (*A*, *k*) and assume that $PR (A, k) \ne \emptyset$ (otherwise, there is nothing to show). Recall that a load distribution $x = (x_{i,c})_{i \in N, c \in C}$ is perfect if ${\bar{x}}_i = \frac{k}{n}$ for all $i \in N$. We first show that there is a perfect load distribution. Let $\{c_1, \ldots , c_k\} \subseteq C$ be a committee providing perfect representation and let $N_1, \ldots , N_k$ be a corresponding partition of *N*. Define load distribution $x^*$ by

$$\begin{aligned} x^*_{i,c_j} = {\left\{ \begin{array}{ll} \frac{k}{n} &{}\text { if }i \in N_j, \\ 0 &{}\text {otherwise.} \end{array}\right. } \end{aligned}$$

It is straightforward to check that $x^*$ is a valid load distribution and that $x^*$ is perfect.

Clearly, a perfect load distribution is an optimal solution for the minimization problem in leximax-Phragmén. It follows that *every* optimal load distribution is perfect. We now show that every perfect load distribution corresponds to a committee providing perfect representation. It then follows that every committee *S* output by leximax-Phragmén provides perfect representation for (*A*, *k*).

Let $x = (x_{i,c})_{i \in N, c \in C}$ be a perfect load distribution and let *S* be the corresponding committee, i.e., $S = \{c \in C: \sum _{i \in N} x_{i,c} = 1\}$.

Define *M* to be an $n \times n$ matrix with rows corresponding to voters and, for each $c \in S$, $\frac{n}{k}$ columns $c^1, c^2, \ldots c^{\frac{n}{k}}$ corresponding to candidate *c*. For $i \in N$ and $c \in S$, define the entry of *M* in row *i* and column $c^j$ (for all $1 \le j \le \frac{n}{k}$) to be $x_{i,c}$. Every row of *M* sums to $\sum _{c \in S} x_{i,c} \frac{n}{k} = \frac{n}{k} {\bar{x}}_i = 1$, and every column of *M* sums to $\sum _{i \in N} x_{i,c} = 1$, so *M* is doubly stochastic. We can now apply the Birkhoff–von Neumann theorem and get that *M* is a convex combination of permutation matrices. Choose a permutation matrix *P* in this convex combination. *P* encodes a bijection between the sets *N* and $\bigcup _{c \in S} \bigcup _{j=1}^{n/k} c^j$. From this bijection, we can extract a partition $\{N(c) \mathrel {:}c \in S\}$ of *N* by defining *N*(*c*) as the set of voters that are mapped to an element of the set $\{c^1, c^2, \ldots c^{\frac{n}{k}}\}$, for each $c \in S$. It is easily verified that this partition satisfies the conditions in Definition 6.2. Therefore, *S* provides perfect representation for (*A*, *k*).

$$\square $$

Since EJR is incompatible with PR (see Example 6.3), leximax-Phragmén fails EJR. However, it satisfies PJR.

#### Theorem 6.11

leximax-Phragmén satisfies PJR.

#### Proof

We introduce one new piece of notation for this proof. For a committee $S\subseteq C$, let $x^S$ be a leximax-optimal load distribution, given that *S* is selected. As usual, we let ${\bar{x}}_i^S = \sum _{c \in S} x_{i,c}^S$.

Consider an instance (*A*, *k*) and a committee *S* output by leximax-Phragmén. Assume that *S* does not satisfy PJR. That is, there exists $\ell >0$ and a group $N^* \subseteq N$ of voters with $|N^*| \ge \ell n/k$, $|\bigcap _{i \in N^*}A_i| \ge \ell$ and $|S \cap (\bigcup _{i \in N^*} A_i)| \le \ell -1$. Note that there must exist a candidate $c^* \in \cap _{i \in N^*}A_i \setminus S$.

The average load among the voters in $N^*$ is

6.3 $$\begin{aligned} \frac{1}{|N^*|}\sum _{i \in N^*} {\bar{x}}_i^S \le \frac{|S \cap (\bigcup _{i \in N^*} A_i)|}{|N^*|} \le \frac{\ell -1}{|N^*|} \le \frac{k}{n}-\frac{1}{|N^*|}. \end{aligned}$$

Further, since the average load among voters in $N^*$ is strictly less than $\frac{k}{n}$ and the total load among all *n* voters is *k*, the average load among voters in $N \setminus N^*$ is strictly greater than $\frac{k}{n}$. In particular, consider a leximax-optimal load distribution $x^S$ and let $i'$ be a voter with maximum load among all voters in $N \backslash N^*$ according to $x^S$. It must be the case that this voter has load ${\bar{x}}_{i'}^S > \frac{k}{n}$.

We can now complete the proof by constructing a committee which has a leximax-smaller vector of voter loads than *S*, contradicting the optimality of *S*. Consider a candidate *c* with $x_{i',c}^S>0$. Such a candidate must exist because ${\bar{x}}_{i'}^S >0$. Consider replacing *c* by $c^*$ to form committee $S'=S \cup \{ c^* \} \setminus \{ c \}$. We construct a valid load distribution *y* for committee $S'$ as follows. Distribute the load of $c^*$ among voters in $N^*$ only in such a way that for each $i \in N^*$, $y_{i,c} \le \max (\frac{k}{n}-{\bar{x}}_i^S,0)$. This is possible because $\sum _{j \in N^*} \max (\frac{k}{n}-{\bar{x}}_j^S,0) \ge \sum _{j \in N^*} (\frac{k}{n}-{\bar{x}}_j^S) \ge 1$, where the last inequality follows from (6.3). Setting $y_{i,c'}=x^S_{i,c'}$ for every voter *i* and every candidate $c' \in S' \cap S$ yields

$$\begin{aligned}&{\bar{y}}_i \le {\bar{x}}_i^S + \max \left( \frac{k}{n}-{\bar{x}}_i^S,0 \right) = \max \left( \frac{k}{n}, {\bar{x}}_i^S \right) \text { for all } i \in N^*\\&{\bar{y}}_{i'} < {\bar{x}}^S_{i'} \,\text {, and} \\&{\bar{y}}_i \le {\bar{x}}_i^S \, \text { for all } i \in N \setminus N^*. \end{aligned}$$

In particular, since ${\bar{x}}^S_{i'}>\frac{k}{n}$ and ${\bar{y}}_i \le \frac{k}{n}$ for all *i* with ${\bar{y}}_i > {\bar{x}}^S_i$, *y* is a leximax-smaller vector of loads than $x^S$, contradicting optimality of *S*. $\square$

#### Remark 6.12

As is the case for seq-Phragmén, leximax-Phragmén also satisfies priceability [40] and therefore IPSC (see Remark 6.7).

#### Corollary 6.13

leximax-Phragmén satisfies JR.

We note that Example 4.1 shows that simply minimizing the maximum voter load (without leximax tie-breaking) does not even yield committees satisfying JR.

### Results for var-Phragmén

The proof of Theorem 6.10 directly applies to var-Phragmén.

#### Theorem 6.14

var-Phragmén satisfies PR.

Unlike leximax-Phragmén, var-Phragmén fails PJR.

#### Example 6.15

Let $C = \{a,b,c,d,e,f,g\}$, $k=6$, and consider the following profile with 100 voters: 67 voters approve $\{a,b,c,d\}$, 12 voters approve $\{e\}$, 11 voters approve $\{f\}$, and 10 voters approve $\{g\}$. Let $N^*$ be the set of voters approving $\{a,b,c,d\}$. We have $|N^*| = 67 \ge 4 \frac{n}{k}$ and $|\bigcap _{i \in N^*} A_i| = 4$. Thus, PJR requires that all four candidates in $\bigcap _{i \in N^*} A_i = \{a,b,c,d\}$ are selected. However, var-Phragmén selects $\{a,b,c,e,f,g\}$.

The previous example also shows that the sequential version of var-Phragmén violates PJR. Finally, we show that var-Phragmén satisfies JR.

#### Theorem 6.16

var-Phragmén satisfies JR.

The proof of Theorem 6.16 can be found in the appendix.

## Relationship to apportionment methods

As mentioned in Sect. 2, the well-studied apportionment problem [8] constitutes a special case of approval-based committee elections. To see this, define a *party-list profile* as a preference profile $A=(A_1, \dots , A_n)$ for which the set *C* of candidates can be partitioned into “parties” $C = P_1 \mathbin {\dot{\cup }} P_2 \mathbin {\dot{\cup }} \ldots \mathbin {\dot{\cup }} P_p$ in such a way that each party $P_j$ contains at least *k* candidates and each voter approves precisely the candidates of one party (i.e., for all $i \in N$, there exists a $j\in \{1,\dots ,p\}$ such that $A_i=P_j$). Each party-list profile *A* can be summarized by a *vote vector* $V_A=(v_1, \ldots , v_p)$, where $v_j= |\{i \in N \mathrel {:}A_i=P_j\}|$ is the total number of votes for party $P_j$. An *apportionment method* is a function that maps a vote vector $V=(v_1, \ldots , v_p)$ and a natural number *k* to a *seat distribution* $z=(z_1, \ldots , z_p) \in \mathbb {N}_0^p$ with $\sum _{j=1}^p z_j = k$. Since vote vectors correspond to party-list profiles, approval-based committee voting rules are generalizations of apportionment methods. As a consequence, every approval-based committee voting rule $\mathcal {R}$ induces an apportionment method $M_{\mathcal {R}}$ [13]: The number $z_j$ of seats that $M_{\mathcal {R}}$ allocates to a party $P_j$ is given by the number $|S \cap P_j|$ of candidates from party $P_j$ that are members of the committee *S* selected by the rule $\mathcal {R}$.

Apportionment methods have been extensively studied by Balinski and Young [8] and Pukelsheim [52]. Three of the most widely-used apportionment methods are

- the *D’Hondt method* (aka *Jefferson method* or *greatest divisors method*),
- the *Sainte-Laguë method* (aka *Webster method* or *major fractions method*), and
- the *largest remainder method* (aka *Hamilton method* or *Hare–Niemeyer method*).

Interestingly, all three apportionment methods are induced by different variants of Phragmén ’s methods: seq-Phragmén and leximax-Phragmén both induce the D’Hondt method [13, 26, 44], var-Phragmén induces the Sainte-Laguë method [13], and Eneström-Phragmén (using the Hare quota $q_H$) induces the largest remainder method [17].^15^

Some of the representation axioms discussed in Sect. 6 have analogies in the apportionment literature: When restricted to party-list profiles, both EJR and PJR (see Definition 6.1) coincide with the requirement that the seat distribution satisfies *lower quota* (i.e., $z_j \ge \lfloor k \frac{v_j}{n} \rfloor$ for all *j*). Therefore, an apportionment method $M_\mathcal {R}$ induced by an approval-based committee voting $\mathcal {R}$ satisfies lower quota whenever $\mathcal {R}$ satisfies PJR. This observation, which was first made by Brill et al. [13], gives rise to an alternative proof for the fact that var-Phragmén fails PJR: var-Phragmén induces the Sainte-Laguë method [13], which is well-known to fail lower quota ([8], p. 130).^16^

Two further properties that are often studied in the apportionment setting are house monotonicity and population monotonicity ([8], p. 117). *House monotonicity* prescribes that no party loses seats when the house size is increased; this directly corresponds to *committee monotonicity* for approval-based committee voting rules. Whereas seq-Phragmén satisfies committee monotonicity by definition, the non-sequential variants leximax-Phragmén and var-Phragmén fail the property. This is implicit already in Phragmén ’s 1896 paper [45], and stated explicitly in the paper by Mora and Oliver [36]; here is a simple example.

### Example 7.1

Let $C = \{a, b, c\}$ and consider the following profile with 10 voters:

$$\begin{aligned} 2 \times \{ a\} \qquad 3 \times \{a,c\} \qquad 3 \times \{b,c\} \qquad 2 \times \{b\} \end{aligned}$$

Both leximax-Phragmén and var-Phragmén select $\{c\}$ for $k=1$ and $\{a,b\}$ for $k=2$.

The D’Hondt method and the Sainte-Laguë method satisfy house monotonicity ([8], p. 100). Consequently, leximax-Phragmén and var-Phragmén satisfy committee monotonicity on party-list profiles. In contrast, the largest remainder method fails house monotonicity and, therefore, Eneström-Phragmén fails committee monotonicity even on party-list profiles.

*Population monotonicity* prescribes that, if the ratio $\frac{v_i}{v_j}$ increases, then it should not be the case that $z_i$ decreases and $z_j$ increases. Population monotonicity is satisfied by the D’Hondt method and the Sainte-Laguë method, but not by the largest remainder method ([8], p. 117). We are not aware of a direct generalization of this property to approval-based committee voting rules; however, it is similar in spirit to *support monotonicity*, introduced by Sánchez-Fernández and Fisteus [54], who showed positive results for seq-Phragmén and leximax-Phragmén.

## Conclusion

We have shown that Phragmén ’s load-balancing methods satisfy interesting representation axioms. In particular, the polynomial-time computable variant seq-Phragmén satisfies PJR. Moreover, both leximax-Phragmén and var-Phragmén satisfy PR and leximax-Phragmén additionally satisfies PJR. Arguably, leximax-Phragmén is the first known example of a “natural” rule satisfying both PR and PJR—the only other rule known to satisfy these two properties is an artificial construct that returns a PR committee if one exists and otherwise runs PAV [56].

Since seq-Phragmén violates EJR, it remains an open problem whether EJR is compatible with committee monotonicity.^17^ Further, the intricate nature of Example 6.9 seems to suggest that instances on which seq-Phragmén violates EJR are rare. It would be interesting to see whether seq-Phragmén satisfies EJR for realistic distributions of preferences and/or for reasonable domain restrictions.^18^ Finally, it would be of great interest to find axiomatic characterizations of Phragmén ’s rules, i.e., to find sets of axiomatic properties that uniquely define leximax-Phragmén, var-Phragmén, seq-Phragmén, and Eneström-Phragmén.

## A Appendix

### A.1 Proof of Theorem 6.16

We first prove a lemma.

#### Lemma A.1

Let $0<\alpha <1$ and $(x_i)_{1\le i \le n}$ be a sequence with $0\le x_i\le \alpha$ for all $i \in \{1, \ldots , n\}$ and $\sum _{i=1}^n x_i = 1$. Then, $\sum _{i=1}^n x_i^2 \le \alpha$.

#### Proof

$$\sum _{i=1}^n x_i^2 \le \sum _{i=1}^n \alpha x_i = \alpha $$. $$\square $$

We can now prove Theorem 6.16.

#### Proof of Theorem 6.16

Consider an instance (*A*, *k*) and a committee *S* output by var-Phragmén. Assume that *S* does not satisfy JR. That is, there exists a group $N^*$ with $|N^*| \ge \frac{n}{k}$, such that $\bigcap _{i \in N^*} A_i \ne \emptyset$ and $|S \cap (\bigcup _{i \in N^*} A_i)| = \emptyset$. Clearly, $|N^*|<n$.

Let $i'$ be a voter with maximum load (i.e., ${\bar{x}}_{i'} \ge {\bar{x}}_i$ for all $i \in N$), and let *c* be a candidate with $x_{i',c}>0$. Such a *c* must exist because the total load on $i'$ is non-zero.

First note that the average load on voters in $N \backslash N^*$ is

$$\begin{aligned}\frac{k}{|N \backslash N^*|} \ge \frac{k}{n-\frac{n}{k}} = \frac{k^2}{kn-n}. \end{aligned}$$

Therefore, since $i'$ is a voter with maximum load, it must be the case that ${\bar{x}}_{i'} \ge \frac{k^2}{kn-n}$. Further, ${\bar{x}}_i = {\bar{x}}_{i'}$ for all voters $i \in N_c$. If this were not the case for some voter *i*, it would be possible to decrease the variance of the load distribution by reducing $x_{i',c}$ by some small amount and increasing $x_{i,c}$ accordingly, thus reducing the difference between the loads on $i'$ and *i* while leaving all other loads unchanged, which reduces the variance.

Let $d \in \bigcap _{i \in N^*} A_i$, and let $T = S \cup \{d\} \setminus \{c\}$. That is, *T* is the committee obtained by starting with *S* and replacing *c* with a candidate approved by all voters in $N^*$. To complete the proof, we consider the effect that this replacement has on the quanity $\sum _{i \in N} {\bar{x}}_i^2$, which is the objective minimized by var-Phragmén.

It is possible to distribute the load of candidate *d* evenly across all (previously unrepresented) voters in $N^*$. Therefore, the addition of *d* contributes at most $\sum _{i \in N^*} \frac{1}{|N^*|^2} = \frac{1}{|N^*|} \le \frac{k}{n}$ to the objective. On the other hand, removing *c* from the committee decreases the objective by

$$\begin{aligned} \sum _{i \in N_c}&({\bar{x}}_i^2 - ({\bar{x}}_i - x_{i,c})^2) = \sum _{i \in N_c} ({\bar{x}}_i^2 - {\bar{x}}_i^2 + 2{\bar{x}}_i x_{i,c} - x_{i,c}^2) = \sum _{i \in N_c} (2 {\bar{x}}_ix_{i,c}-x_{i,c}^2)\\&= \sum _{i \in N_c} (2 {\bar{x}}_{i'}x_{i,c}-x_{i,c}^2) = 2 {\bar{x}}_{i'} - \sum _{i \in N_c} x_{i,c}^2 \ge 2{\bar{x}}_{i'} - {\bar{x}}_{i'} = {\bar{x}}_{i'} \ge \frac{k^2}{kn-n} > \frac{k}{n}, \end{aligned}$$

where the first inequality follows from Lemma A.1. Therefore, replacing *c* by *d* causes a net decrease to the objective, contradicting minimality of the variance of committee *S*. We have thus obtained a contradiction to our assumption that *S* does not provide JR. $\square$

### A.2 seq-Phragmén violates EJR

Table 2 shows the necessary calculations for computing seq-Phragmén in Example 6.9.

**Table 2:** The values $s_c^{(j)}$ (rounded to three decimal places) for each remaining candidate $c \in C$ and each round $j \in \{1, \ldots , 12\}$ in Example 6.9

*c*	$s_c^{(1)}$	$s_c^{(2)}$	$s_c^{(3)}$	$s_c^{(4)}$	$s_c^{(5)}$	$s_c^{(6)}$	$s_c^{(7)}$	$s_c^{(8)}$	$s_c^{(9)}$	$s_c^{(10)}$	$s_c^{(11)}$	$s_c^{(12)}$
$c_1$	0.125	0.163	0.2	0.238	0.275	0.310	0.332	**0.369**	–	–	–	–
$c_2$	0.077	0.119	0.162	0.204	**0.246**	–	–	–	–	–	–	–
$c_3$	**0.05**	–	–	–	–	–	–	–	–	–	–	–
$c_4$	0.05	**0.1**	–	–	–	–	–	–	–	–	–	–
$c_5$	0.05	0.1	**0.15**	–	–	–	–	–	–	–	–	–
$c_6$	0.05	0.1	0.15	**0.2**	–	–	–	–	–	–	–	–
$c_7$	0.05	0.1	0.15	0.2	0.25	**0.275**	–	–	–	–	–	–
$c_8$	0.05	0.1	0.15	0.2	0.25	0.275	**0.325**	–	–	–	–	–
$c_9$	0.05	0.1	0.15	0.2	0.25	0.275	0.325	0.375	**0.388**	–	–	–
$c_{10}$	0.05	0.1	0.15	0.2	0.25	0.275	0.325	0.375	0.388	**0.438**	–	–
$c_{11}$	0.05	0.1	0.15	0.2	0.25	0.275	0.325	0.375	0.388	0.438	**0.488**	–
$c_{12}$	0.05	0.1	0.15	0.2	0.25	0.275	0.325	0.375	0.388	0.438	0.488	**0.538**
*a*	0.25	0.25	0.25	0.25	0.25	0.373	0.373	0.373	0.558	0.558	0.558	0.558
*b*	0.25	0.25	0.25	0.25	0.25	0.373	0.373	0.373	0.558	0.558	0.558	0.558

Entries in bold distinguish the candidate with lowest $s_c^{(j)}$ value (up to tie-breaking) in each round

## Footnotes

1 Proportional Approval Voting is a prominent committee voting rule due to Danish polymath Thorvald N. Thiele [64]. For a detailed comparison between PAV and Phragmén ’s methods, we refer to the works of Janson [26] and Peters and Skowron [40].

2 Two notable studies that predate the conference version of this paper are a survey by Janson [25] (in Swedish) and a paper by Mora and Oliver [36] (in Catalan).

3 A committee voting rule is *committee monotonic* if increasing the committee size results in a winning committee that is a superset of the previously winning committee. An example showing that MES violates committee monotonicity can be found in the survey by Lackner and Skowron [31]. We are not aware of a formal proof that the rules by Aziz et al. [6] fail committee monotonicity, but the way they are defined makes this claim very plausible.

4 The leximax ordering is defined analogously to the more commonly used *leximin* ordering (see, e.g., Moulin [37], Definition 1.1). In the literature, the leximax ordering is referred to as “lexicographic minimax” by Ogryczak [38] and as “lexicographical” by Schmeidler [58].

5 The approximability of leximax-Phragmén has recently been studied by Cevallos and Stewart [18], who showed, in particular, that seq-Phragmén does not offer a constant-factor approximation guarantee.

6 For details on the origin of this rule, see footnote 38 in the paper by Janson [26], who refers to this method as *Phragmén ’s first method*. Sánchez-Fernández et al. [55] refer to the method as *Phragmén-STV*.

7 To see this, consider a cubic graph with an independent set of size *k*. All *k* vertices in the independent set have three outgoing edges and these 3*k* edges must all be distinct, since vertices in an independent set must not be connected via an edge.

8 This result essentially shows that mixed-integer linear programs have solutions of polynomial size.

9 For a general discussion on lexicographic optimization in MILPs, we refer the reader to a paper by Ogryczak and Sliwinski [39] and references therein.

10 This quadratic bound is a very rough estimate and does not use any of the more sophisticated methods for multiplication such as the Schönhage–Strassen algorithm [62] or computing greatest common divisors [11, 33].

11 Aziz et al. [5] have introduced an additional proportionality axiom known as *core stability*. Since core stability is more demanding than EJR, the rules considered in this paper do not satisfy core stability.

12 The incompatibility of PR and EJR was first observed by Sánchez-Fernández et al. [56].

13 Replacing the constraint $|N^*| \ge \ell \frac{n}{k}$ with $|N^*| > \ell \frac{n}{k+1}$ is similar to replacing the Hare quota with the Droop quota in the context of single transferable vote elections (see Sect. 4.3). The condition $|N^*| > \ell \frac{n}{k+1}$ is the best possible here; see the paper by Janson [27].

14 We thank Jannik Peters for pointing out to us that the proof of Peters and Skowron [40] showing that priceability implies PJR can be easily adapted to show that priceability implies IPSC.

15 Under the assumption that there are at least as many seats as there are parties (i.e., $k\ge p$), the optimization variant that maximizes the minimum voter load (see Remark 4.4) induces the *Adams method*. This was remarked by Janson [26] and also follows from Proposition 3.11 of Balinski and Young [8].

16 Indeed, the profile in Example 6.15 is a party-list profile with vote vector (67, 12, 11, 10), for which the Sainte-Laguë method fails lower quota for $k=6$.

17 In the approval-based apportionment setting, where candidates can obtain multiple seats in the committee, EJR and committee monotonicity can be achieved simultaneously [14].

18 Recent experimental work by Bredereck et al. [10] showed that committees satisfying JR very often satisfy EJR as well, supporting the hypothesis that instances for which seq-Phragmén fails EJR are rare. An overview of domain restrictions for approval preferences can be found in the survey by Elkind et al. [20].
